# Characterization of different isogenic mutants derived from improved strains of *Bacillus naganoensis* using Active Hydrogen Bond Network (AHBN) for pullulanase production

**DOI:** 10.1016/j.bbrep.2025.102322

**Published:** 2025-10-29

**Authors:** O.G. Ndochinwa, Qing-Yan Wang, O.C. Amadi, T.N. Nwagu, C.I. Nnamchi, A.N. Moneke

**Affiliations:** a Department of Microbiology Faculty of Biological Science, University of Nigeria, Nsukka, Nigeria; b State Key Laboratory of Biomass Enzyme Technology, National Engineering Research Center for Non Food Biorefinery, Guangxi Academy of Sciences, Nanning, Guangxi, China

**Keywords:** Pullulanase, Active hydrogen bond network, Hydrolysis

## Abstract

This study aimed to improve the thermal and pH stability of pullulanase enzymes through protein engineering. Pullulanase is crucial in various industries, including food, detergent, and textile, as it breaks down starch into simple sugars. Using the active hydrogen bond network (AHBN) method, researchers constructed isogenic mutants of the *Bacillus naganoensis* pullulanase enzyme. The mutants were evaluated for their enzyme activity, pH stability, and thermal stability. Results showed that two mutants, H543R and H543G, exhibited improved biochemical properties compared to the primary mutant E431A. All three mutants had optimal enzyme activity at pH 5.4 and 65 °C. They also showed stability at pH 4.2–5.8 for over 48 h. The T50 experiment revealed that H543R retained 63 % of its enzyme activity at 62.7 °C, while the primary mutant E431A retained 68 %. The TM value experiment showed that H543G and H543R were stable at 65 °C and 64.7 °C, respectively. These findings demonstrate the potential of protein engineering in improving the stability and activity of pullulanase enzymes, which can benefit various industries that rely on starch degradation.

## Highlights

• Engineered pullulanase enzymes show improved thermal and pH stability.
• Protein engineering enhances pullulanase activity and stability for industrial applications.
• Mutant pullulanase enzymes exhibit increased stability and activity at optimal pH and temperature.
• Improved pullulanase enzymes through protein engineering for efficient starch degradation.

## 1 Introduction

The application of enzymes and biocatalysts for various industrial syntheses is rapidly growing [[1], [2], [3], [4]]. Pullulanase (EC 3.2.1.41) is an enzyme that plays a crucial role in starch hydrolysis, specifically by breaking down the α-1,6-glucosidic bonds in starch, pullulan, and related polysaccharides [5,6]. This enzyme is widely utilized in various industries, including food, detergent, biofuel [6], and textile production, as it accelerates the conversion of starch into simple sugars [5]. The enzyme is classified into two types based on substrate specificity: Type I, which acts exclusively on α-1,6-glucosidic bonds in starch and related polysaccharides, and Type II, which targets both α-1,6-glucosidic and α-1,4-glucosidic linkages in many polysaccharides [7]. Among the sources of pullulanase, Bacillus species, especially *Bacillus naganoensis*, are prominent producers, making it a valuable enzyme in industrial applications where the breakdown of complex carbohydrates is required for efficient processes like saccharification, which facilitates the production of fermentable sugars [8].

Despite its widespread industrial use, the application of pullulanase is often limited by its instability at extreme temperatures and pH values, conditions frequently encountered in industrial processes [9]. The enzyme's stability is crucial for maintaining its functionality during large-scale operations, such as starch saccharification, where it works in conjunction with other enzymes like glucoamylase [10]. The instability of pullulanase at high temperatures and acidic pH levels necessitates the development of more robust enzyme variants that can withstand the harsh conditions typically found in industrial settings [9]. This challenge has spurred the need for protein engineering approaches to enhance the thermal and pH stability of pullulanase, ensuring its effectiveness and economic viability in various industrial applications, including bioethanol production, where stable and efficient enzymes are essential.

One promising approach to improving the stability and activity of pullulanase is protein engineering through the Active Hydrogen Bond Network (AHBN) method [[1], [2], [3], [4],^11^]. This method focuses on the modification of the hydrogen bond network within the enzyme, which plays a significant role in stabilizing its structure and facilitating its catalytic activity. In enzymes like pullulanase, the hydrogen bond network connects key residues involved in the enzyme's catalytic function, including a triad of active residues—Asp511, Glu540, and Asp625—within the enzyme's active site [4]. The AHBN method uses computational models to predict and design mutations in these active and functional residues, aiming to increase the enzyme's resistance to thermal denaturation and enhance its performance across a broader pH range. The strategy leverages the enzyme's three-dimensional structure to guide the introduction of mutations that improve its catalytic efficiency, thermostability, and pH stability, which are critical factors for industrial applications [4].

This study applies the AHBN method to engineer isogenic mutants of pullulanase from *Bacillus naganoensis*, with the primary objective of improving the enzyme's thermal and pH stability. The study uses a protein modeling approach to identify potential mutation sites in the pullulanase enzyme, particularly targeting residues near the active site and within the enzyme's overall structure. Several mutants were generated and assessed for their enzyme activity, pH stability, and thermal stability. The results revealed that certain mutants, such as H543R and H543G, exhibited improved properties compared to the wild-type enzyme, showing optimal enzyme activity at pH 5.4 and 65 °C. These mutants also demonstrated higher thermostability and retained significant activity under various pH conditions for extended periods, making them suitable candidates for industrial use in processes like starch saccharification and bioethanol production. The findings highlight the potential of protein engineering to enhance the properties of pullulanase, suggesting that further optimization through the AHBN method could lead to more efficient enzymes for industrial applications, ultimately contributing to the development of more cost-effective and sustainable biocatalysts.

## 2 Materials and methods

### 2.1 Research methodology

#### 2.1.1 Sample collection

The BNPulA324 *Bacillus naganeonsis* used in this experiment was provided by the Key State Laboratory for Biomass Enzyme Technology, National Centre for Research in Non-Food Biorefinery, Guangxi Academy of Sciences, Nanning, Guangxi, China. This organism was stored at 4 °C [4].

#### 2.1.2 Design and construction of isogenic mutants

Strategy and configuration of isogenic mutants: The AHBN was used for the isogenic mutation. Using the swiss model protein modelling server and the pullulanase BAPluA prototype conformation of derived enzyme BAPluA(2WAN), the AHBN of BNPluA derived from Bacillus naganeonsis ATCC53909 was established on an affinity template conformation. The amino acid sequence of oligonucleotides incorporated the significant base transformation as listed in Table 2.

**Table 1:** The original amino acid sequence of pullulanase from *Bacillus naganoensis* (BNPulA324 [4]).

MDGNTTNIVVHYFRPSGDYTDWNLWMWPENGDGAEYDFNQPTDSYGEVASVDIPGNPSQVGIIVRKGNWDAKDIDSDRYIDLSKGHEIWLVQGNSQIFYSEKDAEAAAQPAVSNAYLDASNQVLVKLSQPFTLGEGSSGFTVHDDTANKDIPVTSVSDANQVTAVLAGTFQHIFGGSDWAPDNHNTLLKKVNSNLYQFSGNLPEGNYQYKVALNDSWNNPSYPSDNINLTVPAGGAHVTFSYIPSTHAVYDTINNPNADLQVDSSGVKTDLVAVTLGENPDVSHTLSIQTEDYQAGQVIPRKVLDSSQYYYSGDDLGNTYTKNATTFKVWAPTSTQVNVLLYNSATGAVTKTVPMTASGHGVWEATVNQDLENWYYMYEVTGQGSTRTAVDPYATAIAPNGTRGMIVDLAKTDPAGWESDKHITPKNIEDEVIYEMDVRDFSIDSNSGMKNKGKYLALTEKGTKGPDNVKTGVDSLKQLGITHVQLQPVFAFNSVNENDPTQYNWGYDPRNYNVPEGQYATNANGTTRIKEFKEMVLSLHQDHIGVNMDVVYNHTFATQISDFDKIVPEYYYRTDDAGNYTNGSGTGNEIAAERPMVQKFIIDSLKFWVNEYHVDGFRFDLMALLGKDTMSKAATQLHAIDPGIALYGEPWTGGTSALPADQLLTKGAQKGMGVAVFNDNLRNGLDGSVFDSSAQGFATGATGLTDAIKNGVEGSINDFTASPGETINYVTSHDNYTLWDKIAQSNPNDSEADRIKMDELAQAIVMTSQGIPFMQGGEEMLRTKGGNDNSYNAGDVVNEFDWSRKAQYPDVFNYYSGLIHLRLDHPAFRMTTANEINSHLQFLNSPENTVAYELSDHANKDTWGNIVVIYNPNKTAETINLPSGKWEINATSGKVGESTLGQAEGSVQVPGISMMILHQEVSPSDGK

**Table 2:** Target to improve enzyme activity and stability.

1 MDGNTTNIVV HYFRPSGDYT DWNLWMWPEN GDGAEYDFNQ PTDSYGEVAS
51 VDIPGNPSQV GIIVRKGNWD AKDIDSDRYI DLSKGHEIWL VQGNSQIFYS
101 EKDAEAAAQP AVSNAYLDAS NQVLVKLSQP FTLGEGSSGF TVHDDTANKD
151 IPVTSVSDAN QVTAVLAGTF QHIFGGSDWA PDNHNTLLKK VNSNLYQFSG
201 NLPEGNYQYK VALNDSWNNP SYPSDNINLT VPAGGAHVTF SYIPSTHAVY
251 DTINNPNADL QVDSSGVKTD LVAVTLGENP DVSHTLSIQT EDYQAGQVIP
301 RKVLDSSQYY YSGDDLGNTY TKNATTFKVW APTSTQVNVL LYNSATGAVT
351 KTVPMTASGH GVWEATVNQD LENWYYMYEV TGQGSTRTAV DPYATAIAPN
401 GTRGMIVDLA KTDPAGWESD KHITPKNIEDEVIYEMDVRD FSIDSNSGMK
451 NKGKYLALTE KGTKGPDNVK TGVDSLKQLG ITHVQLQPVF AFNSVNENDP
501 TQYNWGYDPR NYNVPEGQYA TNANGTTRIK EFKEMVLSLH QDHIGVNMDV
551 VYNHTFATQI SDFDKIVPEY YYRTDDAGNY TNGSGTGNEI AAERPMVQKF
601 IIDSLKFWVN EYHVDGFRFD LMALLGKDTM SKAATQLHAI DPGIALYGEP
651 WTGGTSALPA DQLLTKGAQK GMGVAVFNDN LRNGLDGSVF DSSAQGFATG
701 ATGLTDAIKN GVEGSINDFT ASPGETINYV TSHDNYTLWD KIAQSNPNDS
751 EADRIKMDEL AQAIVMTSQG IPFMQGGEEM LRTKGGNDNS YNAGDVVNEF

#### 2.1.3 Site targeting for improved thermostability and enzyme activity

The sites were chosen based on amino acids close to the functional residue. Furthermore, the emphasis on their regular geometries being constrained to a specific value of the dihedral angles (Ø and Ψ) on the Ramachandran plot was considered too. In Table 2, the highlighted letters represent the mutated site targeted to improve the stability of the enzyme. In contrast, the mutated letters within the box represent the mutated site targeted to enhance the enzyme's activity. The mutant modeling and dynamics method was used to predict each mutant, and the difference in amino acid side chains before and after the mutation was compared [4].

### 2.2 Genetic manipulation

The BNPluA324 pullulanase gene (GenBank accession number ATCC53909) from *Bacillus naganoensis* was synthesized by Genescript (Nanjing, China) based on the preferred codon usage of *Escherichia coli* (host gene). Localized or region-specific random mutagenesis mutants were generated through homologous recombination by Genecreate (Wuhan, China). The vector NTI software was used to design the forward and reverse primers of the mutants. To obtain the partial pulA gene, PCR was carried out using Primstar DNA polymerase. The pullulanase BNPulA324 was amplified using the different primers designed above. The genomic DNA from *Bacillus naganoensis* ATCC53909 was prepared and used as a template [4].

#### 2.2.1 Purification of amplified DNA

The purification of amplified DNA was performed using a DNA purification kit. The PCR system comprises the following in μ/L: Template 0.2, primer, up 1.0, primer, down 1.0, DNA polymerase buffer, 5, MgCl2 3, dNTPs 1, DNA polymerase 0.5, H2O 38.3. Purification of the amplified DNA gene was carried out with a DNA purification kit in the following way: first, the column was balanced with balance buffer B.L. (Bacterial lysis buffer) and centrifuged at 12000 rpm for 1 min, then binding buffer P.B. (Phosphate buffer) was added 3 × volume of the total PCR reaction system to 1 volume of the amplified gene and centrifuged at 12000 rpm for 1 min, afterward, the column was washed with wash buffer P.W. (Prewash Solution Concentrate) by addition of 600 μL and centrifuged at 12000 rpm for 1 min, this was repeated for 3 times after which a 50 μL volume of elution buffer was added and centrifuged at 12000 rpm for 1 min to elude the gene.

#### 2.2.2 Restriction enzyme digestion

The reaction for restriction enzyme digestion comprised the following in μ/L: Purified DNA gene 52, restriction enzyme buffer 6, *Nco*I 1, *Xho*I 1. Restriction enzyme digestion was done using *Nco*I and *Xho*I, the fragment was extracted using the gel extraction kit, and the target DNA was linked to the vector (pET-28a+) with T4 ligase through the *Nco*I and *Xho*I site, which introduces a His6-tag at the C-terminus, respectively. The purified DNA fragment extraction was carried out using a gel extraction kit in the following way: First, the DNA fragment was excised from the agarose gel with a clean, sharp scalpel, then the gel slice was weighed, and a 3 × volume of extraction buffer was added to 1 volume of the gel slice, the mixture was incubated at 50 °C until the gel melts in a heating block. The sample was applied to the spin column and centrifugated at 6000 rpm for 1 min. The flow was discarded. A 500 μL volume of extraction buffer was added to the spin column and centrifuged at 12000 rpm for 1 min; again, the flow was discarded. A 750 μL of wash buffer was added to the spin column and centrifuged at 12000 rpm for 1 min. The flow was discarded. A 100 μL elution buffer was added to the spin column to elute the extracted DNA.

The ligation reaction system comprised the following in μ/L: DNA (Pullulanase fragment) 20, DNA (vector) 6, ligation buffer 3, and T4 ligase 1. The ligation reaction was performed using the following steps: A 20 μL DNA volume from the extracted pullulanase fragment was added to the column, followed by addition of 6 μL plasmid vector pET-28(+) volume to the same volume and 3 μL volume of ligation buffer was supplemented to the column. Then 1 μL volume of T4 ligase was added to the column. The column was incubated at a low temperature (−10 °C) overnight.

### 2.3 Expression and purification

The transformation was performed using *Escherichia coli* BL21 (DE3). *Escherichia coli* BL21 (DE3) nurtured pET-28a (recombinant plasmid) was incubated in Lysogeny broth medium, which incorporated 100 μg/ml of kanamycin at 37 °C until the absorbance at 600 reached 0.6. The induction was conducted with isopropyl β-d- thiogalactopyranoside at 30 °C to a final concentration of 0.1 mM for 16 h. Centrifugation was employed to collect the culture supernatants, which were later concentrated with an ultrafiltration device to obtain crude enzymes. The purification process involves subjecting the crude enzyme to the immobilized metal affinity chromatography that uses carboxyl His-tag. The procedure involves the following steps. The matrix was equilibrated with lysis buffer 5 times the column volume. The sample (1 ml of crude enzyme) were loaded into the column. The column was washed with wash buffer 5 times the column volume. The column was eluted with 10 mM, 20 mM, 50 mM, 100 mM, 200 mM, 300 mM, and 400 mM. Fractions with high O.D.>80 values are collected and applied to SDS-PAGE. The column was washed with 5 column volumes of water, then 20 % 3 times the volume of ethanol before storage in a 4–8 °C refrigerator [4].

### 2.4 Pullulanase activity assay

The measurement of pullulanase activity was estimated using 50 mM phosphate-citric buffer (pH = 4.8), while a broader range of pH was established using 50 mM phosphate-citrizinic acid. According to Miller et al. (1994), the experiment involved 1 % (wt/vol) of pullulan used as the substrate with the buffer at 60 °C for 15 min. The amount of enzyme that released 1μ M D glucose from the reducing sugar equals the pullulan used per minute, estimated as one unit of pullulanase activity. The mean of the triplicate experiment was taken as the measurement [4].

### 2.5 Effects of various pH values on enzyme activity

To determine the optimal pH value of isogenic mutants (E431A, H543G, H543R, T586V, T731E, M766F, E835Q, Y736H, S790P, N735G, V551A, N540G)., the enzyme efficiency of pullualanse was calculated in a pH range of 4.2–5.8 (0.2-unit interval was observed) utilizing sodium phosphate citric acid for the mutants. The samples were dissolved in various p H values (pH 4.2–5.8 observing 0.2 unit intervals) in the latency of 50 mM sodium phosphate buffer for mutants to a 0.05 mg/ml protein concentration. They were incubated at 60 °C for 5 min. Therefore, a more comprehensive pH range was used for mutants L605V, F607Y, L681F, and the control represented with E431A∗. The study used sodium phosphate and citrazinic acid buffer, and activity was measured in a pH range of 4.2–8.0(at an interval of 0.2 units). And enzyme efficiency was calculated in 4.2–5.8 p H range (observing interval of 0.2 unit). The samples were dissolved in various pH values (p H 4.2–5.8 observing 0.2 unit intervals) in the latency of 50 mM sodium phosphate buffer for mutants to a 0.05 mg/ml protein concentration. The pullulanase efficiency for 1 % of the enzyme was calculated at 60ᵒC in phosphate citric acid buffer (4.8). The primary activity prior to buffer treatment at 60ᵒC was 100 %.

### 2.6 Investigating the pH stability of the best performed mutants

Residual pullulanase activity was measured after the enzyme was incubated in phosphate citric acid buffer with different pH values, which was used to assess the pH stability. For mutants (E431A, H543G, H543R, T586V), samples were diluted in 50 mM phosphate-citric acid buffer of different pH values (pH 4.2 to 5.8 at an interval of 0.2 unit). In contrast, mutant samples L605V, F607Y, L681F, and E431A ∗ were diluted in 50 mM phosphate-citrizinic acid buffer (pH value 4.2–8 at an interval of 0.2 unit) to a protein concentration of 0.05 mg/ml and incubated at 60 °C for 5 h. After buffer treatment, the residual activity for 1.0 % pullulan was measured at 60 °C in the same buffer (4.8). The initial activity before buffer treatment at 60 °C was 100 %.

### 2.7 Effect of temperature on enzyme efficiency

The connectivity between temperature and activity was measured between 45ᵒC and 70ᵒC (with 5ᵒC intervals), in stipulated buffer (50 mM phosphate-citric acid buffer for mutants E431A, H543G, H543R, T586V, T731E, M766F, E835Q at pH 5.4, while mutants L605V, F607Y, L681F, and E431A ∗was at pH 7.4 using pullulan as substrate). At each temperature, the buffer and pullulan were pre-incubated for 10 min. The reaction was initiated by adding the enzyme and allowed to progress for 15 min. Samples diluted in 50 mM sodium acetate buffer (pH 5.4) at a protein concentration of 0.05 mg/ml were incubated at 60 °C for sometime.

### 2.8 Investigating the thermostability of various mutants

Mutants E431A, H543G, H543R, T586V, L605V, F607Y, L681F, T731E, M766F, E835Q were subjected to heat treatment for 60 min. After heat treatment, the residual activity for 1.0 % pullulan was measured at 60 °C in the same buffer at 10-min intervals starting from 30 min, and the result was used to assess thermos ability. The initial activity before heat treatment was taken as 100 %. The structure of the improved mutant was analyzed by determining the T50 value of the recombinant plasmid, which was measured after heating with a PCR machine. At the same time, the T_M_. value was also analyzed using the circular dichroism spectrum. All values presented in the graphs are the means of different replications.

## 3 Results and discussions

**Fig. SM2:**
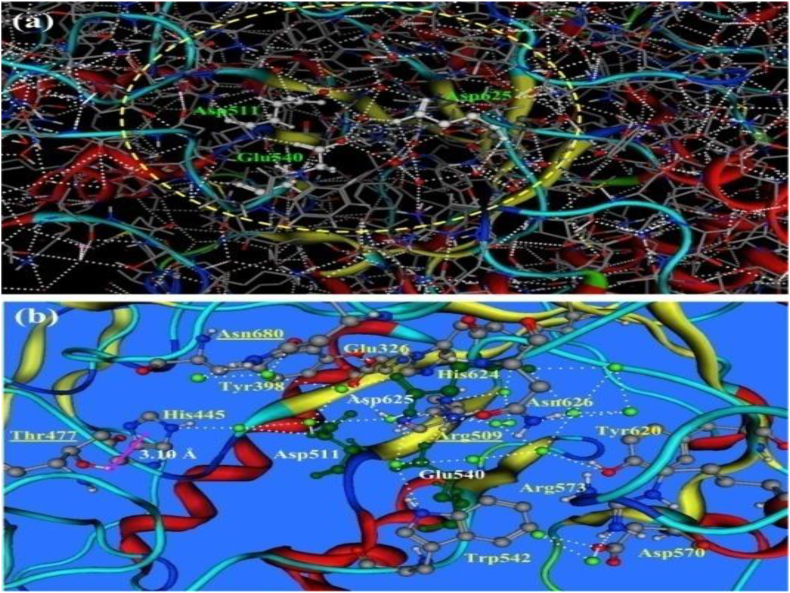
(1A)The complete hydrogen bond network in pullulanase BNPulA324 showing the active residue. (B) Hydrogen bond network of BNPulA324 showing the functional residue (permission to us by Ref. [4]).

Various isogenic mutants of pullulanase enzymes were generated from *Bacillus naganoensis* BNPulA324 based on a well-studied structure of an active hydrogen bond network; these mutants gave rise to a recombinant plasmid as described in the materials and method section [4]. Sites were chosen based on amino acids that were close to the functional residue shown in (Fig. SM2**)**, but the emphasis on their regular geometries being constrained to a specific value of the dihedral angles (Ø and Ψ) on the Ramachandran plot was considered too [4]. Protein function and extracellular production are often influenced by the shape and composition of that particular protein and its interaction with its components. The protein engineering mechanism can serve as an effective tool to increase thermostability, pH stability, catalytic efficiency, and enhance selectivity using these three significant strategies [12,13]: (a) By enlarging the outer surface area charge with numerous hydrogen and ionic interconnections. (b) By enlarging the inflexibility of the refolding area of the protein using extra multiple pair ion network. (c) By enlarging the aquaphobic property of the protein [14].

Data-driven and structure-guided consensus approaches (rational method of mutation) are two primary techniques employed in this research to improve the thermostability and pH stability of mutants derived from different strains of pullulanase enzymes investigated in this study. Chen et al. improved the thermostability of *Bacillus acidopullyticus* using ideas that were developed by other authors, which focused on the identification of flexible regions by determining the high B factor of enzymes, identification of surface residues with solvent accessibility, and use of consensus approach to substitute amino acids [15]. The structure-guided consensus approach employed by different researchers proved that changes in amino acids resulted in the formation of hydrogen bonds, which increased the promotion of van der Waal forces leading to a more thermostable mutant [14,16]. In the study carried out by Wang et al., the substitution of Asp437 with His437 and Asp503 with either Phe503 or Tyr503 (D437H, D503F, or D503Y) resulted in more thermostable mutants because of the formation of more hydrogen bonds that lead to the promotion of van der Waal forces that increased the thermostability of this biocatalyst [4].

The initial stage of the protein engineering process, irrespective of the approach employed, is the probe of that particular protein 3D structure alongside its complex modular architecture; based on this mechanism, Wang et al. studied the active hydrogen bond of BNPulA324 and observed that it was made of three active residues, twelve functional residues and eighteen water molecules [4]. These active hydrogen bond networks transport protons and water molecules to the active site. This movement is responsible for the increased thermostability, pH stability, and catalytic activity of BNPulA324 because enlarging the outer surface charge with extra hydrogen and ionic bonds leads to improved stability (both temperature and pH) and catalytic activity as proven by different authors [4,14,16]. Based on this principle, the mutation occurred at the functional residues inside and outside the Tim barrel (α/β) 8. The data obtained showed that mutations of the functional residues Arg509 and Glu326 situated inside the Tim barrel (α/β) 8 expropriated the bioactivity of the enzyme completely [4].

In contrast, mutations of the functional residues Thr477 and Asn 680 located outside the Tim barrel (α/β) 8 increased the thermostability and pH stability of the pullulanase enzyme more than the wild type [4]. The authors suggested that the change in the polar hydrogen group π bond between Thr477 hydroxyl group (OH) to Asn477 that had an amino group (NH_2_) resulted in shortening the distance between these amino acids; thereby enlarging the inflexibility of the refolding area of the protein [4,14,16]. Furthermore, the saturation mutations at that position showed increased catalytic activity. Double mutations (Asn680Asp and Thr477Asn) at positions 477 and 680 simultaneously showed an extra 0.5U/mL increase than the single mutations [4]. Based on the data derived from this experiment, H543 was observed to be connected to Glu540 located inside the Tim barrel (α/β) 8 and histidine also plays the role of proton donor and acceptor simultaneously. Amino acids associated with the functional residues were also chosen.

**Fig. 1:**
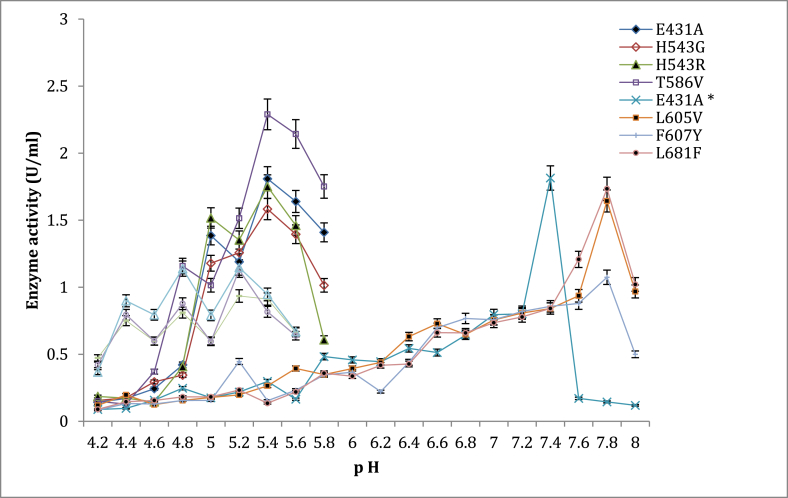
The effect of different pH values on enzyme activity for various mutant with E431A as control at 60 °C.

The results obtained during this work are as follows: “Table 1 represents the sequence of unmuted amino acids of *Bacillus naganoensis* BNPulA324. The active hydrogen bond network (AHBN) method was used for the isogenic mutation [4]. In enzymes, a dimensional network is formed when hydrogen bonds connect the active residues, the functional residues, and conservative water molecules. During the catalytic reaction of hydrolytic enzymes, AHBN is responsible for transporting protons and water molecules; this network is also used to maintain the active and dynamic structure of enzymes. Using the Swiss model protein modeling server and the pullulanase BAPluA template structure of pullulanase BAPluA(2WAN), the active hydrogen bond network of BNPulA324 derived from *Bacillus naganeonsis* ATCC53900 was built based on a homologous model structure. In contrast, Table 2 shows the different targets for improving enzyme stability or activity. The shaded letters, for example, the amino acid in number 431, represented by the letter E, which represents glutamate, was changed to A, which represents alanine targeting improved enzyme stability. On the other hand, the block letter N, which represents the amino acid asparagine at position 504, was changed to glycine to achieve better enzyme activity. Table 3 shows the mutated changes after various sites were chosen, while Table 4 represents the recombinant plasmids selected. Fig. 1 shows the Polymerase chain reaction (PCR) of pullulanase DNA.

**Table 3:** Mutated changes that occurred after various site was chosen.

Target for stability improvement	Target for enzyme activity improvement
E431A	T586V
H543G	T731E
H543R L605V	
F607Y	

L681F	
E835Q	
M766F

**Table 4:** Recombinant plasmids that was generated after insertion.

Numbers	Recombinant plasmids
1	BNPulA E431A in pET-28 a(+)
2	BNPulA H543G in pET-28 a(+)
3	BNPulA H543R in pET-28 a(+)
4	BNPulA T586V in pET-28 a(+)
5	BNPulA L605V in pET-28 a(+)
6	BNPulA F607Y in pET-28 a(+)
7	BNPulA L681F in pET-28 a(+)
8	BNPulA T731E in pET-28 a(+)
9	BNPulA M766F in pET-28 a(+)
10	BNPulA E835Q in pET-28 a(+)

**Fig. 2:**
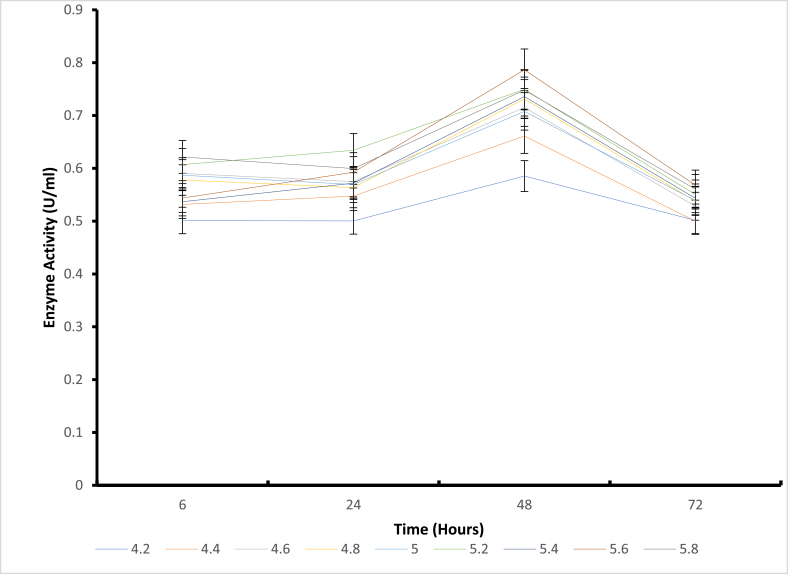
The pH stability of mutant E431A as control at 60 °C temperature.

**Fig. 3:**
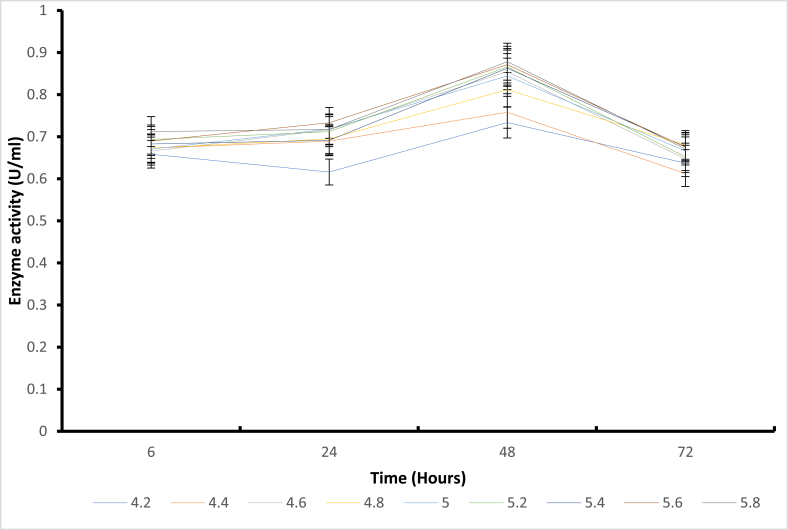
The pH Stability of mutant H543G at 60 °C temperature.

Shifting enzymatic pH optima towards the desired pH values (pH 4.8–6) is crucial for high performance, especially in processes requiring a combination of enzymes (glucoamylase and pullulanase). In China, most bioethanol processes require a combination of glucoamylase and pullulanase for efficient starch hydrolysis. Hence, the study partly focused on ensuring that pH and temperature studies were achieved to accommodate typical fermentation processes in the industry. The pH activity profile for the various mutants is reported in Fig. 1. The mutants T568V, H543G, H543R, E431A control, M766F, T731E, E835Q had their optimum enzyme activity at pH 5.4. Mutants T568V (2.289U/mL), H543R (1.7509U/mL), H543G (1.583U/mL), and E431A (1.809U/mL) (Fig. 3) expressed higher enzyme activity. Using an appropriate buffer system may influence the increase in enzyme activity [17,18]. For pullulanase, it is also recommended to have optimum enzyme activity at pH 4.5 to 6 [4], in some bioethanol processes, these pullulanases are used simultaneously with glucoamylases that have their optimum enzyme activity at a range between pH 4.5 to 6 for starch hydrolysis. Considering this, having the mutant's express high enzyme activity at the desired pH range is advantageous. Research has also revealed that most microbial pullulanases have optimum enzyme activity at weak acidic or neutral pH. Wang et al., reported a pH of 5.0 for BNPulA324 (*Bacillus naganeonsis*) [4], Yang et al., reported a pH of 6.5 for *Bacillus megaterium* [19]*,* Chen et al., reported a pH of 5 for *Bacillus acidopullulyticus* [20]*,* Chen et al., reported pH of 7 for *Paenibacilluslautus* DSM 3035 [15]. In this study, when citrazinic acid and sodium phosphate were used as buffers and the pH values extended (pH 4.2–8), mutant L605V, L681F, and M766F had their optimum enzyme activity at pH 7.8 (Fig. 2), while the control mutant E431A∗had its optimum enzyme activity at pH 7.4. Changes in buffer pH often influence enzyme activity [17,18]. Slightly basic(pH = 8) optimum enzyme activity was reported for *Geobacillus thermoleovorans* pullulanase by Nisha and Satyanarayana [21]. Studies by Chen et al., also showed pH 7 for *Paenibacillus lautis* DSM 3035 pullulanase [15]. Although the mutants in the study expressed high enzyme activity (pH = 7), they were screened out or not recommended for use to accommodate glucoamylase and pullulanase simultaneous application.

**Fig. 4:**
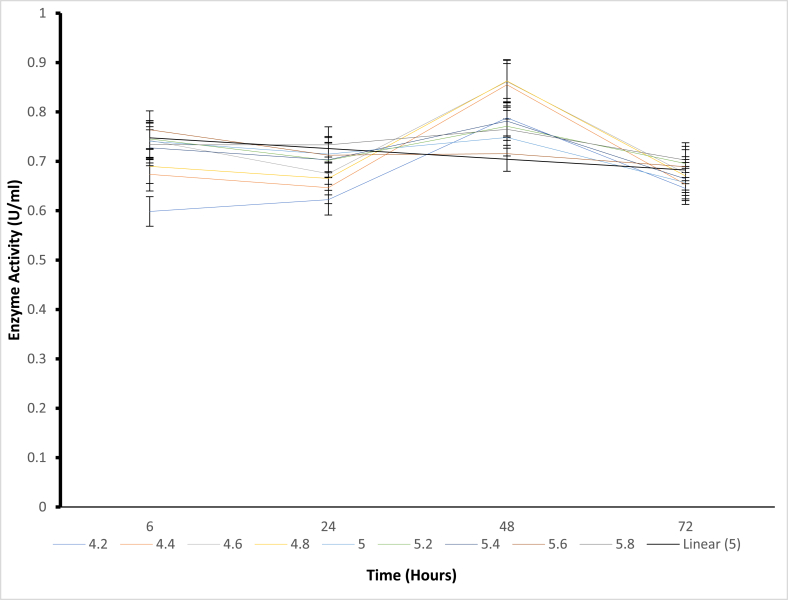
The pH stability of mutant H543R at 60 °C temperature.

**Fig. 5:**
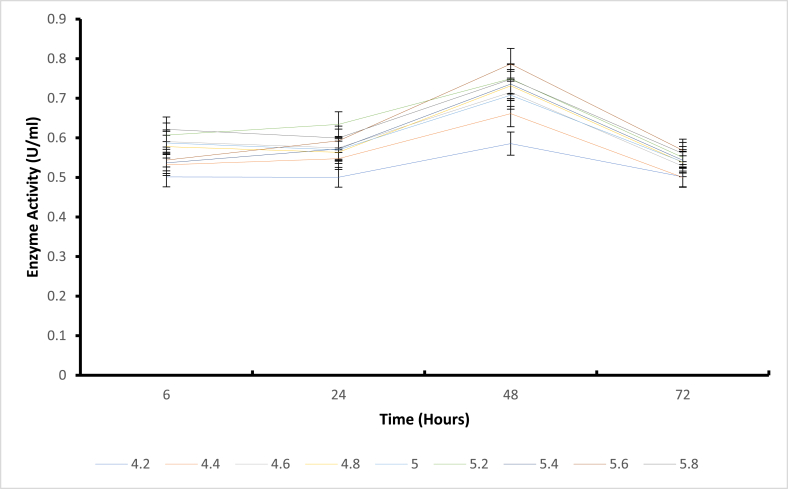
The pH stability of mutant T586V at 60 °C temperature.

Structural stability is essential for maintaining functional conformations when adverse environmental conditions (temperature, pressure, pH, presence of solvents, and salts) are encountered [4]. Stability is also an important property that affects the structure and function of macromolecules and determines biological fitness [22]. Based on these reports, stability appears to be a significant limitation when using enzymes in non-natural environments [23]. Enzymes are extensively studied to understand the trade-off between activity and stability and to engineer more tolerant variants. Several stabilization strategies have been proposed for pH-dependent denaturation. For example, the electrostatic interactions of the charged residues were suggested as a critical factor of adaptation to pH. Again, the substitution of basic by acidic residues was used to improve the charge balance and stability at low pH, and vice versa. It has also been reported that asparagines and glutamine deamidate in an alkaline medium can destabilize the protein structure [24]. On the other hand, mutation of these residues was suggested to improve stability at extreme alkaline conditions [25]. Finally, alteration of pKa values of the critical residues; either manually selected [26] or known to be catalytically important [27] were implemented to design pH stability profiles. Additional studies have shown that introducing amino acid residues with ionizable side chains can modulate pH-dependent protein stability. Regarding the pH stability (Fig. 2, Fig. 3, Fig. 4, Fig. 5) for all mutants in the present study, it was observed that mutant H543G, H543R, T586V, and control E431A consistently expressed an increase in enzyme activity. Mutants H543G and H543R had optimum stability at pH 5.4 and maintained 50 % and 43 % during the 48-h incubation period. This experiment was carried out to determine how stable the mutants can be across a range of pH 4.2–5.8 within a certain period (6–72 h) of time, as shown in Fig. 2, Fig. 3, Fig. 4, Fig. 5, [28].

**Fig. 6:**
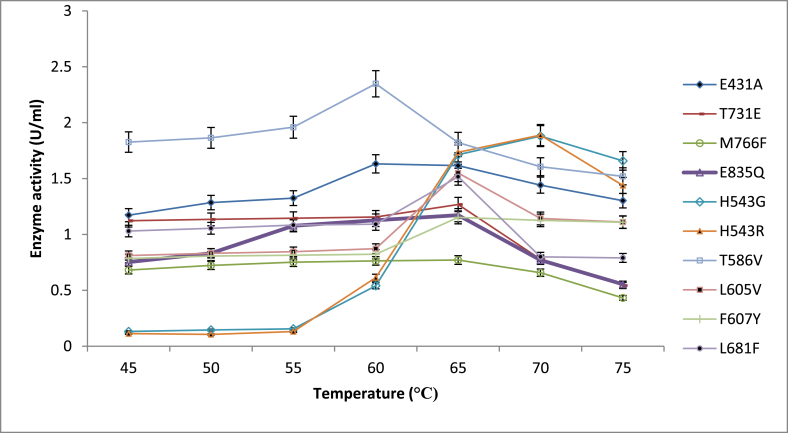
Shows the effect of different temperature values on enzyme activity for various mutant with E431A as control.

**Fig. 7:**
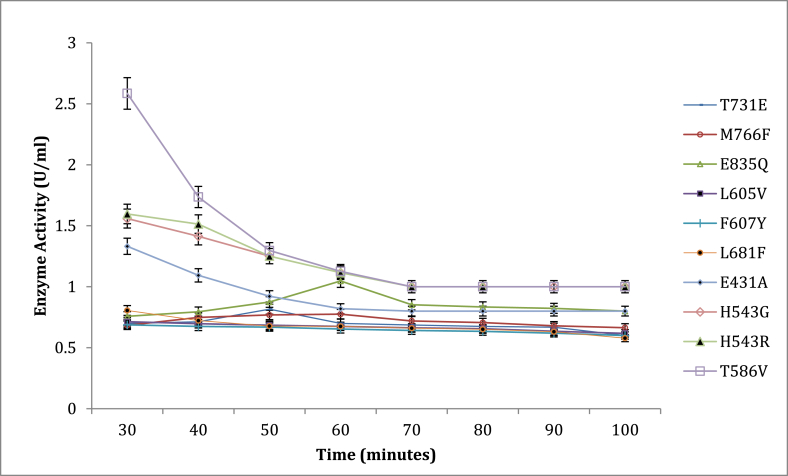
The thermostability of mutant E431A(control), H543R, H543G and T586V at 65 °C while other mutants was at 60 °C.

Effect of temperature on enzyme activity (Fig. 6) showed optimum temperature for mutants T568V, N735G, N504G, N553P, and V551A at 60 °C while E431A, L605V, F607V, L681F, T731E, M766F, E835Q, had their optimum enzyme activity at 65 °C. Mutants H543G and H543R had their optimum enzyme activity at 70 °C. The thermostability experiment (Fig. 7) showed that optimum thermostability was observed at 65 °C for mutants H543G and H543R, and they retained about 38 % and 63 % of their activity, respectively, at 65 °C. Again, pullulanase, which displays optimum temperature and thermostability at 60°C–65 °C, is advantageous for reasons given earlier (glucoamylase exhibits its maximum capacity). Glucoamylases are used together with pullulanase for an effective and total breakdown of starch materials resulting in increased sugars and, consequently, ethanol; it was, therefore, essential to have pullulanase with appropriate pH values and increased temperatures that will complement glucoamylases used in starch hydrolysis [4]. Furthermore, retaining activity and stability after 48-h are used in the starch saccharification process. Mutants from this experiment that had higher enzyme activity and pH stability (H543G and H543R) than the control mutant (E431A), were used for the T_M_ and T50 experiments. There are many studies on thermal stability since most reactions are at elevated temperatures for improved productivity and the exclusion of microbial contamination [4,14,29]. The saccharification process, usually carried out at 60 °C temperature and over 48-h, is a critical step in the starch conversion process. This experiment is essential because thermal stability during saccharification cannot be overemphasized. Enzymes tailored to possess this capability are promising candidates in any starch processing industry [29].

**Fig. 8:**
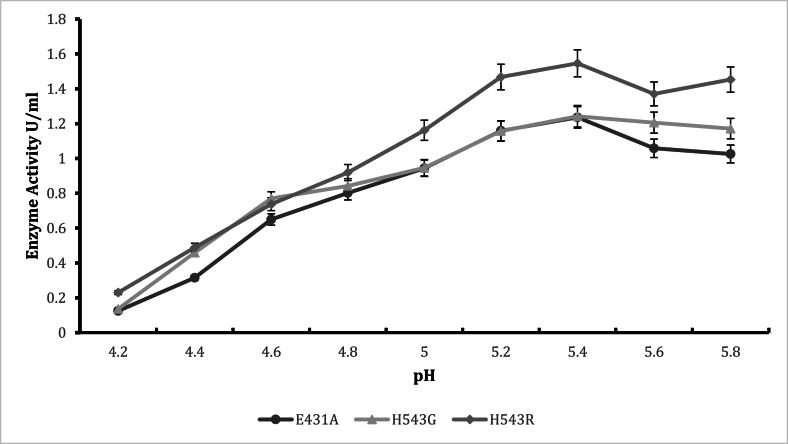
The confirmation experiment for the effect of pH on enzyme activity on mutant H543G, H543R and E431A as control at temperature 60 °C.

**Fig. 9:**
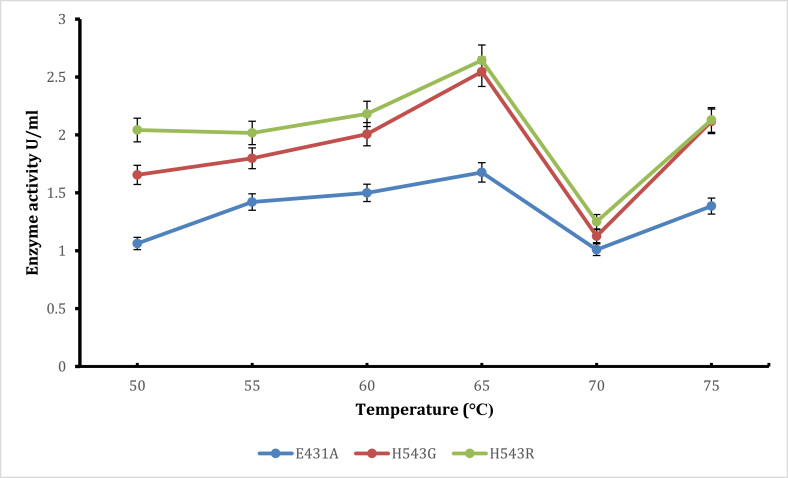
The Confirmation experiment on effect of different temperature values on mutant H543G, H543R and E431A as control.

**Fig. 10:**
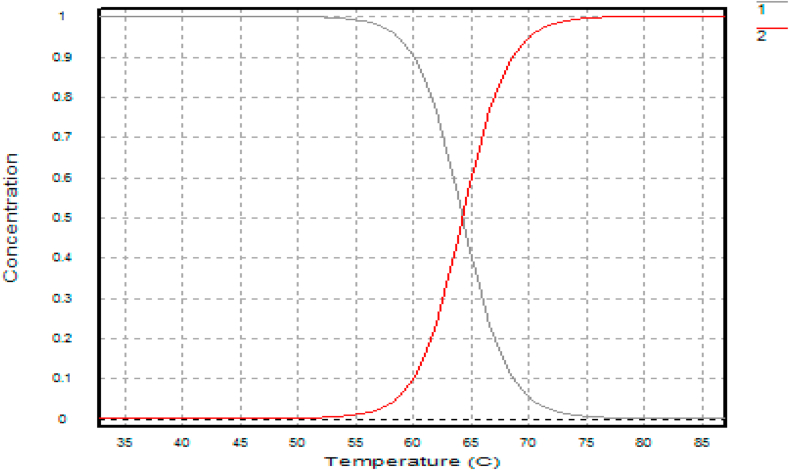
The T_M_ value of mutant E431A (Control).

**Fig. 11:**
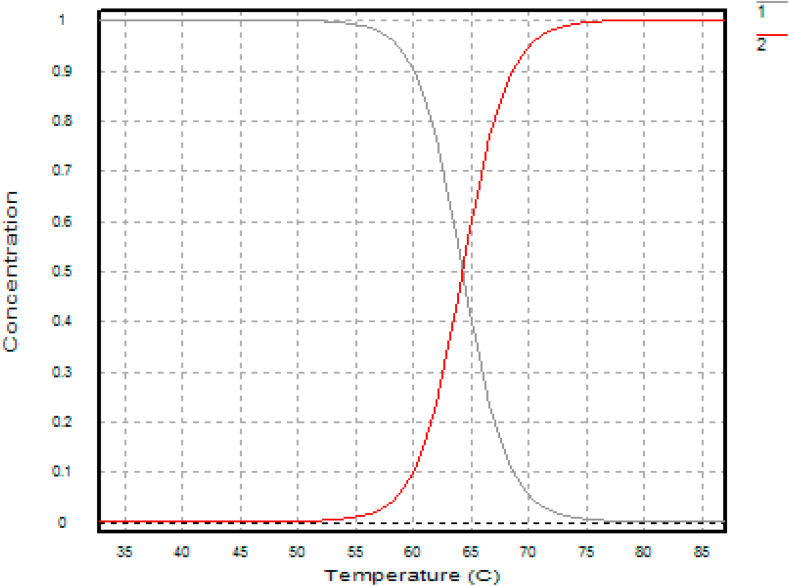
The T_M_ value of mutant H543R.

**Fig. 12:**
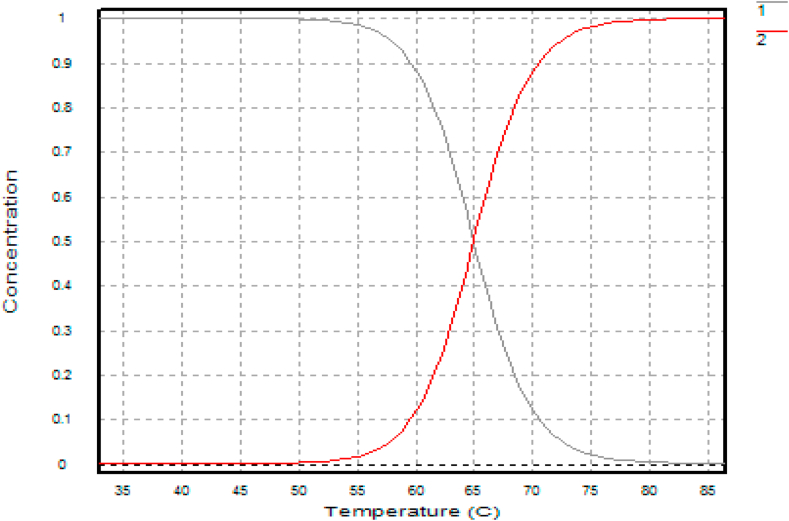
The T_M_ value of mutant H543G.

**Fig. 13:**
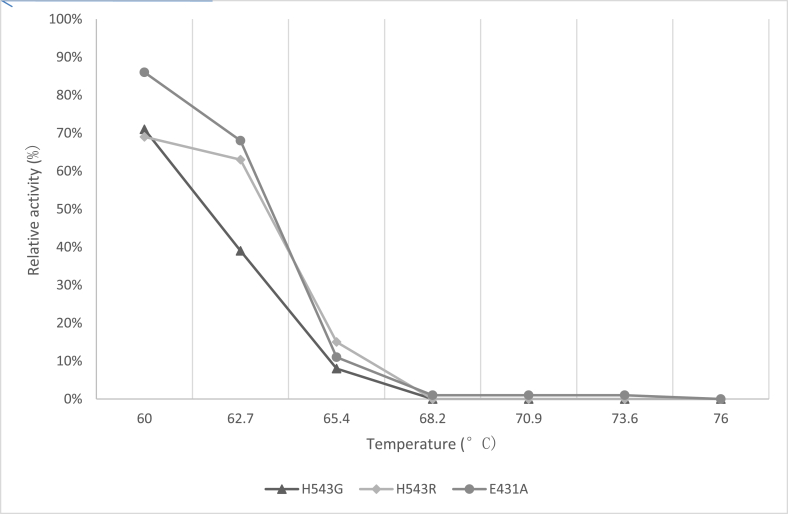
The T50 values of mutant E431A, H543G and H543R at different temperature ranges.

Confirmatory experiments (Fig. 8, Fig. 9) were carried out for the effect of pH and temperature. The best-performing mutants (H543G and H543R) were selected for this experiment because their enzyme activity was higher than that of the control mutant E431A. Both mutants had their optimum pH and temperature at pH 5.4 and 65 °C. The control had the most minor enzyme activity at the same pH value. Confirmation results are significant in this experiment since they help establish concrete evidence of the previous results. The saccharification process of starch molecules involves glucoamylase (acts on α 1, -4 glucosidic linkage) and pullulanase (acts on α 1, -6 glucosidic linkages) simultaneously to ensure proper breakdown of starch components [30]. It is essential to tailor pullulanase with biochemical characteristics (optimum pH and temperature, thermostability, and pH stability) with glucoamylases to ensure proper conversion of starch molecules. Since mutants E431A, H543G, and H543R had better enzyme activity at increased temperatures and were more thermostable than the other mutants, their melting points were determined, and the results are shown in Fig. 10, Fig. 11, Fig. 12. This experiment used circular dichroism, and the value was pinpointed to the point where the black and red lines met. The T_M_ value for the control mutant E431A is 64.3 °C (Fig. 10). The T_M_ value of mutant H543R is 64.7 °C (Fig. 11), while the T_M_ value of mutant H543G is 65 °C (Fig. 12), and the value was pinpointed where the black line and the red line met. Fig. 13 shows the T50 values of mutants E431A, H543G, and H543R at different temperature ranges. The mutant E431A (control) retained 68 % relative activity, while mutants H543R and H543G retained 63 % and 38 %, respectively.

The highlight of this research was the determination of the T_M_ and T50 values of these mutants, which are essential biochemical properties for pullulanase used in the saccharification process. Most authors have reported various biochemical properties of pullulanase, but none has expressly noted the T50 and T_M_ values. Data generated from this experiment contradicts Xu et al. [31]. They suggested that *Escherichia coli* has a low expression system and requires the addition of surfactants like Triton X-100, CHAPS, Tween 80, or sodium taurocholate to solubilize the enzymes [31]. This reaction increased the catalytic activity of the biocatalyst, but the case is different in this research. Although Triton X-100 was added to help blow up the cells of *Escherichia coli BL21* (DE3), there was an insignificant difference in these mutants catalytic or enzyme activity. Screening of the various recombinant plasmid mutants was done as described in the materials and methods section. The results shown indicated that only two recombinant plasmid mutants out of the twenty-eight different recombinant plasmid mutants BNPulA**H543G** in pET-28a (+) and BNPulA**H543R** in pET-28a (+) respectively exhibited higher biochemical properties (enzyme activity) than the primary mutant BNPulA **E431A** in pET28a (+). These two recombinant pullulanases had various optimum enzyme activities at pH 5.4 in 20 mM phosphate citric acid buffer. Recombinant plasmids mutants BNPulA E431A in pET-28a (+), BNPulA H543G in pET- 28a (+) and BNPulA H543R in pET-28a (+) had their optimum enzyme activity at temperature 65 °C. The Mutation at the H543 position showed an increase of 0.5U/ml in enzyme activity at pH 5.4 compared to the previous study that had optimum pH 5.0, and an optimum temperature of 65 °C in contrast to the wild type in the last survey that had 55 °C [4]. The T50 and T_M_ experiments were also conducted to confirm the thermostability and catalytic activity. The T50 experiment showed that the enzyme retained 63 % of its enzyme activity at 62.7 °C while the T_M_ was 64.7 °C. The pH stability experiment showed stable enzyme activity after 24 h, with a slight increase in activity after 48-h. The concentration of these enzymes was also determined; BNPulA H543G in pET-28a (+) and BNPulA H543R in pET-28a (+) had 5 mg/ml concentration, while BNPulA E431A in pET-28a (+) had 2 mg/ml concentration.

## 4 Conclusion

Recombinant pullulanases from BNPulA324, which includes BNPulA324H543G in pET −28a (+) and BNPulA324H543R in p ET-28a (+), were discovered in this study. After cloning and biochemical characterization, these enzymes were observed to record an optimum pH value of 5.4 and an optimum temperature value of 65 °C. They were also stable in pH values of 4.2–5.8 for over 48 h, with a slight increase in enzyme activity. The protein concentration was estimated at 5 mg/ml for mutant H543R and 2 mg/ml for mutant H543G. The T50 value was ascertained to be 62.7 °C, and the T_M_ value was recorded as 64.7 °C and 65 °C for mutant H543G and H543R, respectively. These properties made them valid candidates in various starch processing industries, including the Chinese biofuel industry. Furthermore, future mutations are recommended at position H543.

## Contribution to knowledge

This research has truly unlocked the potential of engineering enzymes using active hydrogen bond networks, in order to obtain biocatalysts with desirable biochemical properties that could be utilized in various industrial activities.

## Declaration of competing interest

The authors declare that they have no known competing financial interests or personal relationships that could have appeared to influence the work reported in this paper.

## Figures and Tables

**Fig. SM1:**
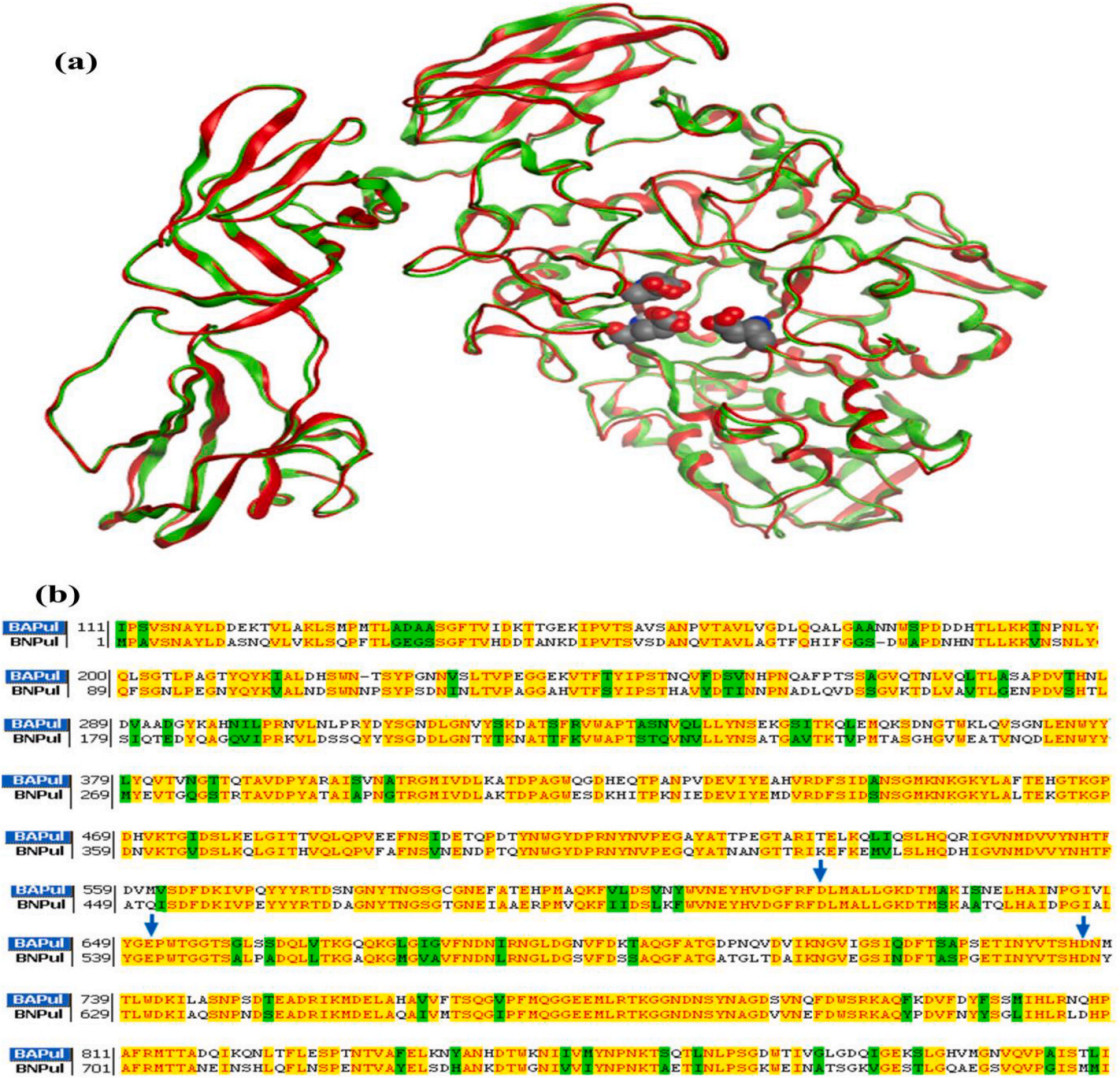
The affinity 3D conformation of pullulanase with code BNPulA324 derived from Bacillus naganeonsis was established consolidating on the researched conformation of pullulanase derived from BAPulA (2WAN). Aspartic acid 511, glutamic acid 540 and aspartic acid 625 located at the ball render that represents the active residual. Model pullulanase (represented in red color) and template pullulanase (represented in green color) constitutes the two peptide backbone which are reliable, Represents the model pullulanase BNPulA324 and template pullulanase BAPulA (2WAN) alignment. It also shows 65.93 % affinity of the two pullulanase (permission to use by Ref. [4]).

**Table:** 

	60	62.7	65.4	68.2	70.9		76
H543G	71 %	39 %	8 %	0 %	0 %		0 %
H543R	69 %	63 %	15 %	0 %	0 %		0 %
E431A	86 %	68 %	11 %	1 %	1 %	1 %	0 %

## Data Availability

Data will be made available on request.

## APPENDICES

### Homology model of pullulanase BNPulA324

The affinity model of pullulanase derived from Bacillus naganoensis with code ATCC53909 had 65.93 % identity conformation with pullulanase derived from Bacillus acidophilus with code (pdb 2WAN). This affinity is the maximum compared to other structures of pullulanase in NCBI protein bank. This made room for pullulanase 2WAN to be most preferred as the template for affinity structure construction using software (Swiss model protein modelling server. Fig SM1A illustrates the 3D structural alignment that involves the template and model pullulanse and Fig SM1B illustrates the alignment of two pullulanase. In figure SM1C, the two structures that consists of the template and model were duly represented consistently, signifying the affinity model structure of pullulanase derived from Bacillus naganoensis with code BNPulA324 is dependable.

### Active hydrogen bond network (AHBN) in pullulanase

Biological software which include SYBYL-X 2.1 (http://www.tripos.com/) could be used to establish the hydrogen bond network in protein. The complete hydrogen bond network in pullulanaseBNPulA324 (including crystal water molecules) is shown in Fig SM2. In Fig SM2A; oxygen and nitrogen (which are electronegative atoms) are connected to the hydrogen atom that are represented by the white dash line, with the amino acids residuals in the polar hydrogen atoms (backbone and sidechains), and water molecules. It could be complicated to study the entire hydrogen network of proteins, meanwhile the active hydrogen bond network is independent and easy to comprehend. The active residual that consists of aspartic acid 511, glutamic acid 540, and aspartic acid 625, surrounded at an active region known as the catalysis triad, which is situated ahead of (α/β)8 TIM Barrel are responsible for the active site of BNPulA324.Tracing of this network begins with the active residual, aspartic acid 511, glutamic acid 540, with aspartic 625, functional residual that consists of glutamic acid 326, tyrosine 398, histidine 445, threonine 477, arginine 509, threonine 509, tyrosine 542, aspartic acid 570, arginine 573,tyrosin 620, histidine 624, asparagine 626, asparagine 680, and eighteen water molecules (watermolecule 311, watermolecule 602,watermolecule 603,watermolecule 604, watermolecule 605, watermolecule 630,watermolecule659,watermolecule662,watermolecule724,watermolecule727,watermolecule729,watermolecule730,watermolecule731,watermolecule732,watermolecule734,watermolecule 786 watermolecule 788, watermolecule 952) interconnected by bond formed with hydrogen atoms. 2 to 4 bonds formed by hydrogen atoms are crosslinked with each water molecules in an active hydrogen bond network [4].
